# Academia, Journal Publishing and the Bio-Medical Industry

**DOI:** 10.4103/0973-1229.32144

**Published:** 2007

**Authors:** Ajai R. Singh, Shakuntala A. Singh

**Affiliations:** Editors, The Mens Sana Monographs, India. mensanamonographs@yahoo.co.uk

The collaboration between academia, journal publishing and the biomedical industry has come to stay. We may rave or rant, but that's the reality. The problem is how to regulate it so it's win-win for all concerned.

We suggest the parties concerned look to their long-term interests if they are, and want to continue to remain, long-term players. Even their short-term interests may not be jeopardized if they do.

Well, how?

Let the man of medicine, and academia in general, accept that profit per se is not a dirty word. And all business in health care is not necessarily shady.

Let academia realise that getting pliant researchers and producing favourable results may get some dollars initially, but will reduce research credibility eventually. And then, the institution will get lesser funds, as well as poorer quality of researchers and academicians.

Let journal editors and publishers realise that doctored clinical trial publishing may fill pages and get ads/supplement sponsors etc., but will ultimately result in reduced credibility for what's published in its pages. For, you cannot publish retractions all the time. And corrections and counter viewpoints are all fine. But ultimately, everyone knows it's so much better to work so one has to publish correction and retractions as less as possible.

And publishing counter viewpoints that take the wind out of the sales of a published paper is all right occasionally. But, if it happens regularly, it does reflect on the credibility and quality of the papers published in the journal. And its editorial board and peer review process. Similarly, not publishing negative results may help get friendly with sponsors, but patients will fall sick. And lawsuits will be slapped. And the original publication, its authors and the journal, will be known and talked of, as well.

With the increasing number of lawsuits against industry, it will not be long before the pattern of pliant clinical trial research in pliant journals will be deciphered and exposed.

Then, the journal's credibility must take a severe beating.

Let the pharma industry realise that manipulation of results does not actually serve it's own welfare. For ultimately, patients will not get really well and the drug will fall by the way side.

So it serves the interest of all concerned to work for getting grants (academia), or ads/supplement sponsorship (journals), or earning profits (industry), by really working for patient welfare.

## No Other Worthwhile Option

They have no real worthwhile option.

Carry out rigorous research. Produce drugs that work. Reject ones that don't. Thank the researcher for giving negative results if the drug doesn't work. For he saves the drug company millions in fictitious campaigning.

Just think of it. If you produce drugs that work, how can physicians resist prescribing it? And how can profits not flow?

Then all the measures to hype up the product are valid. And all the profits that accrue legitimate.

If journals publish only rigorously well-designed studies and also negative studies, how will they not establish credentials? And how will they not get increased revenue if their credentials are impeccable?

The worry that funds will not flow in from sponsors is misplaced. For, sponsors will be forced to support credible journals for their own survival. Why? Simple because publication there ensures their own credibility. It's better journals work so the power of money does not bend them, but gets bent to serve the interests of scientific integrity and patient welfare. It's not as difficult as it may seem, if we give up our cynicism and are ready to sustain ourselves for the protracted good fight.

Then greater Academia-Industry connect would be worthwhile. And welcome.

Lesser lawsuits from disgruntled patients and patient-right advocates would result.

Lesser patient dissatisfaction and greater esteem, both for the pharma companies whose profits may be soaring today, but reputation is at a real ebb; and for academia, whose coffers maybe filling up with sponsors' funds, but credibility is taking a real all-round beating.

As also for journal editors and publishers, whose image is still preserved in the general public and general medical reader, but is already showing signs of wilting under a scorching media gaze and that of ethically conscious peers.

## Enlightened Self-Interest

So, we think it is really in their own self-interest they all work for patient welfare. And do replicable studies.

For these two are the bedrock of any worthwhile research.

And promote vigorously those drugs/processes/implements that stand these two tests.

And till they do, pressure must be mounted on them so they cannot but see what really is in their own welfare.

But at no step should we overbalance and paint all Academia-Industry connect as bad and suspect every move of collaboration. That only makes them immune to our earnest efforts for them to see reason.

Neither does it help to paint pharma/medical implement manufacturer as the villain and academia as the good-boy-gone-bad due to evil company. That too makes both defensive and aggressive.

And when people get defensive/aggressive, they may pursue their misdirected agenda all the more forcefully. Or decide to just shut their ears.

Neither of which is useful to the campaign of ethical practice in medicine.

All this, provided this discussion is, in some way, directed to achieving that goal at some time or the other.

## What Can You Do?

In the meanwhile, as we wait for all stake holders to see reason and their own enlightened self-interest, how do we resolve the issue?

The problem has to be tackled at various levels. By statutory bodies, Researchers, Medical journalists, editors, activists, end-users.

But what can the prescribing doctor do? He can start with the following:

1. Always see, and demand, conflict of interest revelations of authors, especially of those who write Clinical Practice Guidelines, report drug trials, write drug reviews. As also of CME presenters of such drugs. Many spokespersons of pharma help palm off marketing manoeuvres as evidence based medicine.
2. Always verify whether the trial reports of a drug touted are as per revised ICMJE guidelines, in which researchers have access to full trial data and control over publication of results.
  That they were involved all through in the write-up, right from first draft to the final text.
  This is important to prevent manipulation of data, publishing doctored results and ghost writing which is pretty rampant and serves as another marketing manoeuvre masquerading as evidence based medicine.
3. Never be impressed by glossy one-page write-ups by pharma reps, but ask for the original paper where it is published, and see whether point 1 and 2 are addressed.
4. Enjoy the fare at pharma sponsored CME, if you cannot resist, but keep your critical antennae up. If some drug is being hailed as the new miracle drug, just note that few months back some other drug was being similarly hailed. Important breakthroughs are welcome, but are few and far in-between.

## Take Home

- All Pharma/industry is not bad.
- All Academia is not bad either.
- There are, however, numerous areas of concern in their connect.
- All can be resolved if patient welfare and scientific evidence, especially replicable studies, become the litmus test. And they look to their long-term interests.
- That's the Win-Win for all players concerned.

